# EHR Interoperability Experiences Reported by Family Physicians

**DOI:** 10.1001/jamanetworkopen.2025.42460

**Published:** 2025-11-13

**Authors:** Jordan Everson, Julia Adler-Milstein, Robert L. Phillips, Andrew W. Bazemore, Vaishali Patel

**Affiliations:** 1 Office of the National Coordinator for Health Information Technology and Assistant Secretary for Technology Policy, Washington, District of Columbia; 2 Department of Family Medicine, Georgetown University, Washington, District of Columbia; 3 Center for Clinical Informatics and Improvement Research, Department of Medicine, University of California, San Francisco; 4 Center for Professionalism and Value in Health Care, American Board of Family Medicine, Washington, District of Columbia

## Abstract

**Importance:**

Improving the interoperability of electronic health records (EHRs) is a core health care policy goal and an important enabler of high-quality continuous care.

**Objective:**

To describe the extent to which primary care physicians have ideal interoperability experiences and simulate the potential impact of interventions to improve those experiences.

**Design, Setting, and Participants:**

This cross-sectional study leveraged the 2024 American Board of Family Medicine Continuous Certification Questionnaire, fielded November 2023 to October 2024, to conduct a survey of family physicians about their interoperability experiences. To estimate the impact of hypothetical interventions, responses to individual survey questions were artificially modified and the impact on rates of reported ideal interoperability experience were observed. Data were analyzed from October 2024 to January 2025.

**Exposures:**

Principal practice size, ownership, EHR system used, gender, age, urban vs rural location, and physician-reported percentage of patient panel who are vulnerable and who are value-based care patients.

**Main Outcomes and Measures:**

Outcomes of interest were reported ideal interoperability experiences for 6 data types and 5 document types, with ideal interoperability experience defined as often automatically obtaining information, easily finding that information, and easily comparing or reconciling it, all within the EHR.

**Results:**

A total of 8122 family physicians responded to the survey (100% response rate), with 2336 respondents (29%) working in independent medical practice. Physicians’ reported ideal interoperability experiences varied by data type, ranging from 19% for encounter documents to 8% for test results from outside hospitals or health systems. Illustrative simulations showed that interventions that increased the proportion of physicians who reported often automatically obtaining medications by 20 percentage points increased the proportion of physicians reporting ideal interoperability experiences for medications by 1 percentage point. In multivariate regression models examining ideal interoperability experiences by practice characteristics, physicians with vulnerable patients representing more than half of their panel were less likely to report ideal interoperability experiences for primary care notes and consultation reports from independent practices (odds ratio, 0.66; 95% CI, 0.48-0.91) but not for medications (odds ratio, 0.96; 95% CI, 0.73-1.27).

**Conclusions and Relevance:**

In this cross-sectional study, family physicians indicated that despite public policies designed to ensure that EHRs support standards-based data exchange and incentivize clinicians to engage in exchange, these policies had not fully translated into widespread interoperability for all use cases. Collaborative efforts that simplify interoperability and more robustly support data standardization and quality are needed to improve system-wide interoperability.

## Key Points

**Question:**

What percentage of primary care physicians report experiencing ideal interoperability, defined as information is available, easy to find, and easy to use?

**Findings:**

In this cross-sectional study using survey data from 8122 family physicians, the percentage of physicians experiencing ideal interoperability varied across data types, ranging from 8% for test results from health systems to 19% for encounter documents.

**Meaning:**

Few physicians report ideal interoperability experiences, indicating a continued need for multiple cross-cutting interventions to improve the availability and quality of clinical data across health care delivery organizations.

## Teaser

This cross-sectional study describes opinions of family physicians on interoperability of electronic health records (EHRs) and simulates the potential impact of interventions to improve interoperability.

## Introduction

Improving the interoperability of electronic health records (EHRs)—the ability of systems to exchange and use information from other systems—is viewed as essential to enabling coordinated care, population health, fully informed medical decision-making, and artificial intelligence in health care.^1,2,3^ Given their role in care continuity and coordination, primary care physicians (PCPs) are directly affected by their EHR’s interoperability and have a unique view on the extent to which interoperability is progressing toward an ideal user experience similar to other sectors.^4,5,6^ PCPs’ experiences are far from ideal: interoperability is the most desired improvement to EHRs reported by clinicians, and recent studies show modest satisfaction with current EHR interoperability among family physicians.^3,7,8^ That dissatisfaction is supported by related reports showing that integration is the least common component of interoperability reported by both hospitals and physicians^9,10^; that few data elements were standardized with matching codes across health care organizations^11,12^; that quality issues, including missing data, inhibit the use of data^9,13^; and that it is difficult to make data useful for analytics.^14^

Addressing these issues in ways that directly achieve ideal experiences for physicians requires ensuring that distinct forms of interoperability are in place. These align with the 4 levels of interoperability defined by the Healthcare Information and Management Systems Society: foundational (establishing interconnectivity), structural (the format, syntax, and organization of data), semantic (use of standardized definitions to establish shared meaning), and organizational (governance, social, and policy).^15^ Furthermore, it is important that each level is approached with a range of data types and diverse organizations across the health care delivery system in mind.

Specific information on how physicians experience accessing and using health information from outside organizations could help technology developers, organizations, and policymakers better target initiatives to address key challenges related to each level of interoperability and how EHRs use interoperable data to support physicians.^8^ To that end, we conducted a national survey on physicians’ interoperability experiences to address 3 questions. First, what proportion of PCPs report ideal interoperability experiences with their EHR system when used across organizations, and which interoperability levels exhibit the greatest gaps? Second, how do these experiences vary across different data and document types and across physician and practice characteristics? Third, how would hypothetical interventions to improve each level of interoperability affect the proportion of physicians reporting ideal interoperability experiences?

## Methods

### Survey Development and Administration

The American Board of Family Medicine (ABFM) annually requires completion of a Continuous Certification Questionnaire (CCQ) for all Family Medicine Diplomates completing a certification cycle. The CCQ is a required part of the ABFM certification process for more than 106 000 family physicians and by design has a 100% response rate with required responses for each question. Family physicians take this web-based survey on a rolling basis every year based on their initial year of certification, offering a nationally representative sample. Existing questions on use of health information technology and interoperability were updated for the 2024 CCQ. To reduce response burden, the survey questions included in this study were divided into 2 halves and each was randomly assigned to half of respondents, resulting in minimal differences in respondent characteristics. This study followed the Strengthening the Reporting of Observational Studies in Epidemiology (STROBE) reporting guideline for cross-sectional studies and was granted exempt status by an official from the Assistant Secretary for Technology Policy with a waiver of informed consent because of the use of secondary data.

Survey questions on EHRs drew on 2 sources: previously fielded and tested questions and new questions generated based on 20 interviews with family physicians.^7,16^ Key insights from these interviews included: (1) physicians conceptualize ideal interoperability as readily accessing information from other organizations within their EHR and that the information is easy to find, use, and compare with internal information, and this conceptualization drove our measurement of ideal interoperability experience; (2) physicians described readily accessing information as automatically obtaining it within their EHR rather than relying on manual efforts to track down and scan or import information; (3) physicians’ ideal interoperability experiences vary by data type, requiring a tailored measurement approach; and (4) physicians could readily report on the extent to which their current EHR supported seamlessly obtaining, easily finding, and easily comparing and reconciling information. The study team developed new items to capture these insights. Four focus groups with family physicians, followed by a technical expert panel, were used to cognitively test the survey items and assess its validity. The survey was administered from November 2023 to October 2024. Survey responses were imported to Stata software version 15 (StataCorp) for analyses.

### Outcomes

The focal outcomes were the extent of ideal interoperability experiences by data type. The survey included questions about respondents’ experiences with distinct levels of interoperability for 6 types of discrete data. For each question, response options were often, sometimes, rarely, never, and do not know. For 4 types of discrete data (medications, allergies, problems, and immunizations), ideal interoperability experience was captured as the respondent reporting: (1) that they often “automatically electronically obtain this information from outside organizations in [their] EHR” (enabled by foundational interoperability), (2) that their EHR often “makes it easy to find” that information (enabled by structural interoperability), and (3) that their EHR often “makes it easy to reconcile the differences between information from outside organizations and our internal data” (enabled by semantic interoperability).

The survey also included questions for 2 types of laboratory results. The first 2 questions were the same as above. The third was that information often “includes [laboratory results] ordered by other physicians for my patients,” and the fourth was that the EHR often “makes it easy to compare the results from outside organizations and our internal results”.

The survey asked respondents about their experiences with 5 types of documents (independent primary care notes or specialist consultation reports, health system primary care notes or specialist consultation reports, encounters, independent imaging center reports, and health system imaging reports). Interoperability experience was captured as the respondent reporting that (1) they often “automatically electronically obtain this document from outside organizations in [their] EHR,” (enabled by foundational interoperability) (2) it is often “easy to find the document in [their] EHR,” (enabled by semantic interoperability such that the system knows what type of document it has received) and (3) it is often “easy to find the information that [they are] looking” for within a document (enabled by structural interoperability, such that the system knows what part of the document is, for example, an assessment and plan).

### Individual and Practice Characteristics

We captured characteristics relevant to use of interoperable EHRs: respondent’s age and gender, the practice’s EHR developer (ie, eClinicalWorks, Allscripts, Cerner, Epic, NextGen, Practice Fusion, unknown, athenahealth, or other), principal practice size (1-5, 6-20, >20 clinicians practicing at the site), ownership (ie, academic health center or faculty practice, safety net [Federally Qualified Health Center, Rural Health Clinic, nonfederal government clinic]; independently owned medical practice, or other [managed care or health maintenance organization, Indian Health Service, federal, workplace clinic, or other]), location, physician-reported percentage of patient panel who are vulnerable, and value-based payment participation.

### Statistical Analysis

To identify the proportion of physicians who reported ideal interoperability experiences, we ordered responses to survey questions into a hierarchy that reflected the levels of interoperability: readily obtained, easy to find, and easy to compare with internal information. We then calculated the percentage of respondents who reported that they often experience each level of interoperability (the most positive response option) and the preceding level for each type of data. For example, we calculated the percentage of respondents who reported both that they often automatically obtain medications from outside organizations in their primary EHR and that their primary EHR often made it easy to find those medications. We then calculated the percentage who reported often experiencing one level of interoperability who then reported not often experiencing the subsequent level (eg, they often automatically obtained medications, but they were never easy to find in the EHR). We examined differences in each level of interoperability across data types using McNemar test.

We next constructed multivariable logistic regression models to assess how characteristics of the responding physician, their practice, and EHR were associated with the likelihood of an ideal interoperability experience. The dependent variable in these models was ideal interoperability experience (ie, responding often to all levels for that data type).

Finally, we created illustrative simulations of the impact of potential technology innovations and policies on physicians’ ideal interoperability experiences to identify whether targeting a specific level of interoperability could result in substantial improvement or whether across-the-board improvement is needed. These simulations are intended as illustrative of the dynamics involved in improving ideal interoperability experiences and not intended to reflect the impacts of specific innovations. Nevertheless, the simulations are motivated by real-world efforts, including the Trusted Exchange Framework and Common Agreement, which primarily impacts foundational and organizational interoperability^6^; implementation of technical solutions such as Epic System’s Happy Together that display information from outside organizations in line with internal information by leveraging structural interoperability^17^; and implementation of the US Core Data for Interoperability within Health Level 7 Fast Healthcare Interoperability Resources (FHIR) Standard, which would improve semantic interoperability.^18^ We conducted simulations with 2 alternative assumptions. First, we randomly assigned family physicians to have an improved experience of each level of interoperability without a change to other levels of interoperability. Second, we increased the proportion of physicians who were in the ideal interoperability experience group for each level of interoperability by 20 percentage points and assumed these physicians had ideal interoperability experience on subsequent levels of interoperability at the same rate as family physicians who reported that experience on the survey. *P* values were 2-sided, and statistical significance was set at *P* < .05. Additional details are available in the eMethods in Supplement 1.

## Results

A total of 8122 family physicians (mean [SD] age, 4869 [60%] years; 3737 [46%] women) responded to the CCQ and questions about interoperability. Among these, 2840 family physicians (35%) primarily practiced in a site owned by a health system or hospital while 2336 physicians (29%) primarily practiced in an independent practice (eTable 1 in Supplement 1). Furthermore, 3428 family physicians (42%) used Epic as their primary EHR, and 1561 physicians (19%) used an EHR not listed in the survey or that was used by fewer than 100 respondents.

### Interoperability Experience by Structured Data Type

**Figure 1.**
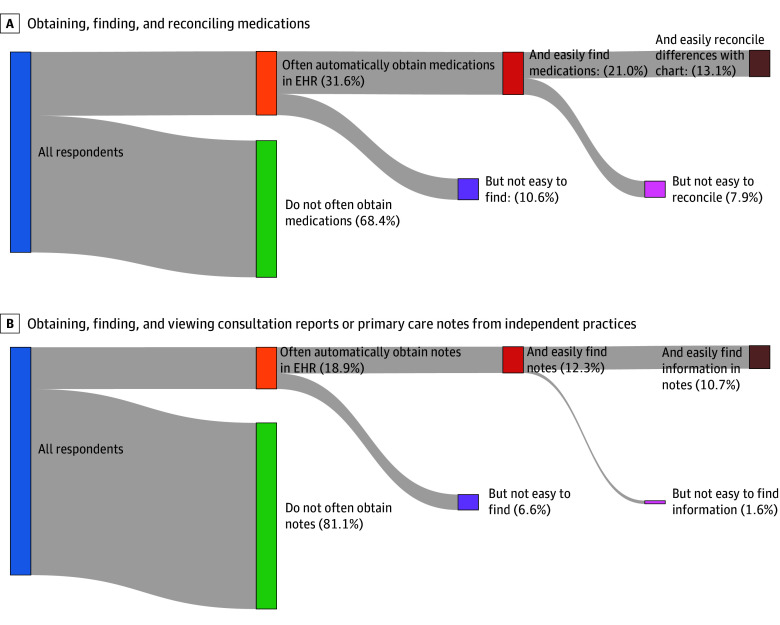
Ideal Interoperability Experience for Medications and Consultation Reports or Primary Care Notes From Independent Practices A total 4107 physicians reported on their experience with medications and 4015 reported on their experience with notes from independent practices. Respondents skipped questions on whether their electronic health record (EHR) made it easy to find and to use information if they reported that they did not know if they obtained that information in their EHR or never obtained it in their EHR. Respondents indicating do not know or never were coded as not often automatically obtaining information in their EHR and retained in the denominator.

Approximately half of the overall sample (4081 respondents [50%]) received questions about their experience with discrete data. Approximately one-third (1291 respondents [32%]) of these reported often automatically obtaining medications in their EHR (Figure 1). Of these, 67% said that their EHR made it easy to find medication information, resulting in 863 family physicians (21%) who often automatically obtained medications in their EHR and reported it was often easy to find medication information. Of these, 62% reported it was often easy to reconcile information in their EHR. Thus, 536 family physicians (13%) of reported an ideal interoperability experience for medications. The proportion of physicians who reported ideal interoperability experience varied by data type (Table 1): 696 respondents (17%) for immunizations and just 318 respondents (8%) for test results from hospitals or health systems, the lowest percentage of any data type.

**Table 1.** Ideal Interoperability Experience for Multiple Data Types

Data type	Respondents, No. (%)
Medications (n = 4107)	
Often automatically obtain in EHR	1291 (32)
And often easy to find information	863 (21)
And often easy to reconcile differences	536 (13)
Allergies (n = 4107)	
Often automatically obtain in EHR	957 (23)^a^
And often easy to find information	699 (17)^a^
And often easy to reconcile differences	563 (14)
Problems (n = 4107)	
Often automatically obtain in EHR	954 (23)^a^
And often easy to find information	677 (16)^a^
And often easy to reconcile differences	514 (13)^b^
Immunizations (from all potential sources, including state immunization registry) (n = 4107)	
Often automatically obtain in EHR	1166 (28)^a^
And often easy to find information	875 (21)
And often easy to reconcile differences	696 (17)^a^
Test results from commercial laboratories (Reference) (n = 4107)	
Often automatically obtain In EHR	1343 (33)
And often easy to find information	776 (19)
And often obtain information ordered by other physicians for my patients	465 (11)
And often easy to compare results from outside organizations	318 (8)
Test results from outside hospitals or health systems (n = 4006)	
Often automatically obtain In EHR	741 (19)^a^
And often easy to find information	453 (11)^a^
And often obtain information ordered by other physicians for my patients	403 (10)^a^
And often easy to compare results from outside organizations	301 (8)
Primary care notes and specialist consultation reports from independent practices (Reference) (n = 4015)	
Often automatically obtain In EHR	760 (19)
And often easy to find document in EHR	492 (12)
And often easy to find information within the document	431 (11)
Health system primary care notes / specialist consult reports (n = 4022)	
Often automatically obtain In EHR	783 (19)
And often easy to find document in EHR	537 (13)^b^
And often easy to find information within the document	465 (12)
Encounters (n = 4022)	
Often automatically obtain In EHR	1290 (32)^a^
And often easy to find document in EHR	898 (22)^a^
And often easy to find information within the document	752 (19)^a^
Independent imaging center reports (n = 3991)	
Often automatically obtain In EHR	1039 (26)^a^
And often easy to find document in EHR	678 (17)^a^
And often easy to find information within the document	579 (15)^a^
Health system imaging reports (n = 4015)	
Often automatically obtain In EHR	959 (24)^a^
And often easy to find document in EHR	673 (17)^a^
And often easy to find information within the document	600 (15)^a^

Abbreviation: EHR, electronic health record.

^a^ Each column indicates the percent of respondents that responded often to that question and prior questions about that data type. For each data and document type, respondents skipped questions on whether information was easy to find and use if they reported that they did not know whether they obtained that information in their EHR or never obtained it in their EHR. Respondents indicating “Don’t know” or “Never” were coded as not often automatically obtaining information in their EHR and retained in the denominator.

^b^ McNemar tests whether the percentage of respondents that reported each level of interoperability was different from the reference category: *P* < .05.

### Interoperability Experience by Document Type

An additional half of respondents (4015 respondents [50%]) received questions about their experience with documents. Only 760 respondents (19%) indicated that they often automatically obtained outside notes from independent practices in their EHR. Approximately two-thirds (63%) of these reported that their EHR often made it easy to find these documents, resulting in 492 family physicians (12%) who often automatically obtained notes from independent practices and often easily found them. Of these physicians, 88% also reported easily finding information within notes. Thus, 431 family physicians (11%) reported an ideal interoperability experience overall for notes from independent practices in their EHR, the lowest percentage among surveyed document types. The document type with the highest percentage of physicians reporting an ideal interoperability experience was for encounter documents, with 752 respondents (19%) of physicians reporting such experience.

The modal response for most items was sometimes, indicating widespread partial progress and that between 2% and 11% of respondents indicated do not know for each question (eTable 2 and eTable 3 in Supplement 1). In total, between 40% and 45% of family physicians reported sometimes or often experiencing ideal interoperability for medications, allergies, problems, immunizations, and test results (eTable 4 in Supplement 1). Between 49% and 61% of physicians reported sometimes or often experiencing ideal interoperability for encounters (2475 physicians [61%]), notes and consultation reports, and imaging reports from health systems and imaging centers (1957 physicians [49%]).

### Factors Associated With Ideal Interoperability Experiences

**Figure 2.**
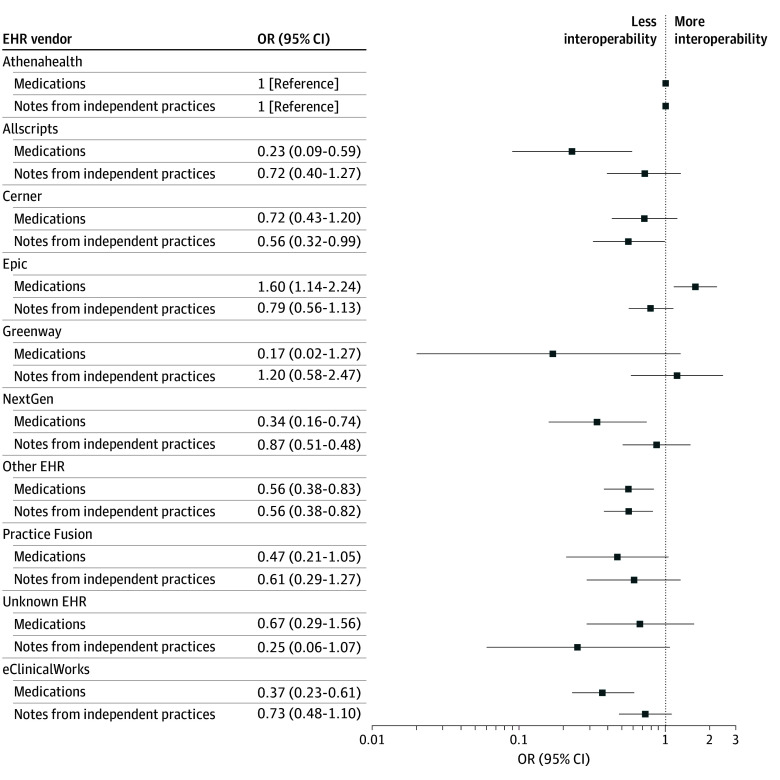
Multivariate Logistic Regression Model for Association of Electronic Health Record (EHR) System With Ideal Interoperability Experience for Medications and Notes From Independent Practices

**Figure 3.**
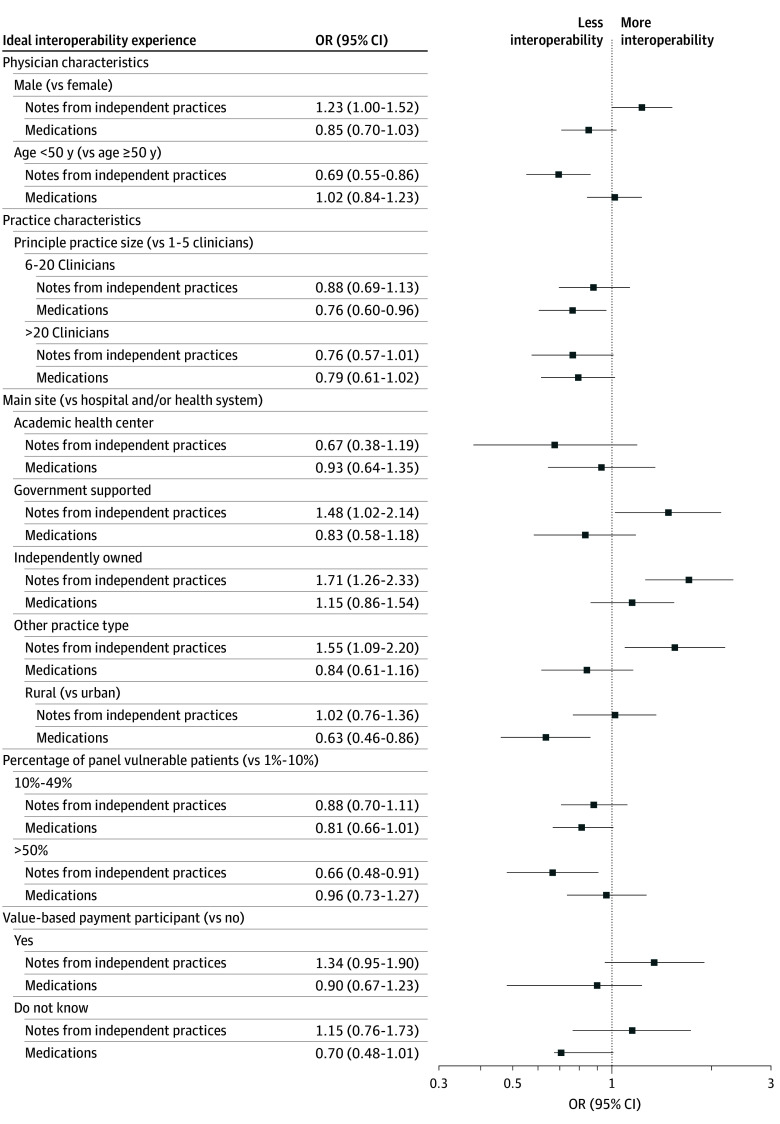
Multivariate Logistic Regression Model for Association of Physician Characteristics With Ideal Interoperability Experience for Medications and Notes From Independent Practices A total of 36 respondents indicated that their gender was other or that they preferred not to answer, and all of these respondents did not report ideal interoperability for medications and were therefore excluded from the model.

Patterns of associations between independent variables and outcomes differed substantially in models estimating ideal interoperability experiences for medications and notes from independent practices (Figure 2). Compared with users of athenahealth, users of Epic were substantially more likely to report ideal interoperability experiences for medications (odds ratio [OR], 1.60; 95% CI, 1.14-2.24) and users of other vendors, including eClinicalWorks (OR, 0.37; 95% CI, 0.23-0.61), NextGen (OR, 0.34; 95% CI, 0.16-0.74), and Allscripts (OR, 0.23; 95% CI, 0.09-0.59) were substantially less likely. However, EHR developer was not associated with the likelihood of ideal interoperability experiences for notes from independent practices. Family physicians in rural areas were less likely to report ideal interoperability experiences for medications (OR, 0.63; 95% CI, 0.46-0.86), but not for notes from independent practices (Figure 3). Physicians who reported a larger proportion of their panel from vulnerable groups were less likely to report ideal interoperability experiences for notes from independent practices (OR, 0.66; 95% CI, 0.48-0.91) but were no more or less likely to report ideal interoperability experiences for medications. Results for all data types are presented in eTable 5 and eTable 6 in Supplement 1 and indicate some consistency (eg, rural location associated with lower rates of ideal interoperability for 3 of 5 discrete data types) but also highlight important idiosyncrasies.

### Simulation of Technical and Policy Interventions

Table 2 illustrates the simulated impacts for each effort, alone and in combination, focused on medications. We found that when impactful interventions occur for 1 level of interoperability in isolation, the overall change in physicians reporting ideal interoperability experience was minimal: a 20–percentage point increase in automatically obtaining medications in the EHR resulted in just a 1–percentage point increase in ideal interoperability experiences being reported by physicians. When these interventions were assumed to have knock-on effects on subsequent levels of interoperability, the impact of each intervention on the proportion of physicians with ideal interoperability experiences was larger but still suppressed by more than half. In either case, these findings suggest that substantial increases in the proportion of physicians with ideal interoperability experiences would require improvements in multiple or all levels of interoperability.

**Table 2.** Potential Impact of Interventions to Improve Interoperability of Medication Information

Intervention	Respondents, % (95% CI)					
	Isolated improvement^a^			Interrelated improvement^b^		
	Often automatically obtain in EHR	And often easily find information	And often easy to reconcile differences (ideal interoperability)	Often automatically obtain in EHR	And often easily find information	And often easy to reconcile differences (ideal interoperability)
As reported	32 (30-32)	21 (20-22)	13 (12-14)	32 (30-33)	21 (20-22)	13 (12-14)
Individual interventions						
TEFCA-like improvement that increases the proportion of physicians that automatically obtain medications by 20 percentage points	52 (50-53)	24 (23-25)	14 (13-15)	52 (50-53)	35 (33-36)	21 (20-23)
Usability improvement that increases the proportion of physicians who report it is easy to find automatically obtained information by 20 percentage points	32 (30-33)	25 (24-27)	14 (13-15)	32 (30-33)	28 (26-29)	17 (16-18)
Data quality or standards-adherence improvement that increases the proportion of physicians who report it often easy to reconcile different medications received in their EHR by 20 percentage points.	32 (30-33)	21 (20-22)	16 (15-17)	32 (30-33)	21 (20-23)	17 (16-19)
Combination of interventions						
Both TEFCA-like and usability improvement	52 (50-53)	40 (39-41)	15 (14-16)	52 (50-53)	45 (44-47)	28 (27-29)
Both TEFCA-like and data quality improvement	52 (50-53)	24 (23-25)	16 (15-17)	52 (49-55)	34 (33-36)	28 (27-30)
Both usability and data quality improvement	32 (30-33)	25 (24-26)	20 (19-21)	32 (30-33)	28 (26-29)	22 (21-24)
All 3 improvements	52 (50-53)	40 (39-41)	28 (27-29)	52 (50-53)	45 (44-47)	37 (36-39)

Abbreviations: EHR, electronic health record; TEFCA, Trusted Exchange Framework and Common Agreement.

^a^ Isolated improvements refer to a scenario wherein improvement on 1 level of interoperability did not impact their responses to other levels of interoperability. CIs for the as-reported values were derived from survey data and student *t* tests. CIs for each isolated improvement intervention were estimated using 1000 bootstrap samples drawn with replacement.

^b^ Interrelated improvements assume that following an improvement to 1 level of interoperability, the likelihood that the respondents experiences ideal interoperability on subsequent levels matches the likelihood of individuals who reported the improved state on the survey. CIs for the as-reported values were derived from survey data and student *t* tests. CIs for the interrelated improvement interventions were calculated via Markov chain with a sample of 4081 and 10 000 simulations.

## Discussion

This cross-sectional study describes a timely national survey with a 100% response rate from family physicians on their interoperability experiences. Less than one-third of family medicine physicians indicated that they often automatically obtained information from outside organizations in their EHR across data types. Even physicians who routinely obtained information automatically in their EHR frequently indicated that information was not easy to find or use within their EHR, indicating either a lack of structural or semantic interoperability or systems that do not leverage interoperability to make data usable. We also did not find a specific group of physicians with more universally positive experiences from which we could learn how to scale such achievement. This may be because any given physician’s experience with interoperability depends not only on their system but also the systems of all other organizations at which their patients received care.

It would be reasonable to expect greater reported availability of information given the substantial volume of exchange reported by prominent networks^19^ and the longstanding incentives for hospitals and clinicians to use their EHR to exchange information created by the Medicare Promoting Interoperability Program and Promoting Interoperability component of the Medicare Incentive Payment System.^20^ However, it is possible that existing networks only reach a limited subset of family physicians or that the volume of useful exchange is smaller than public statistics indicate. Incentives from Promoting Interoperability may have had limited effect because they are small and only apply to hospitals and a subset of clinicians. Newer policies that incorporate the Trusted Exchange Framework and Common Agreement, which establishes a nationwide floor for information exchange across organizations and networks, could increase rates of foundational interoperability by growing its participant base. However, our evidence, coupled with other recent evidence showing that connectivity between organizations alone is not sufficient to result in improved care,^21^ highlight that policies that impact connectivity alone and do not address other facets of interoperability may lead to limited use of information and diminished impact on patients.

Today, certified EHRs used by most hospitals and physicians are required to have the capability to support data standards for the data types assessed here and to support the reconciliation and incorporation of outside information into a patient’s record.^18,22^ However, use of certified EHRs does not necessarily mean that the data recorded in them is always high quality, usable, and consistently adheres to the standards supported by certified EHRs or that receiving EHRs are leveraging interoperable data to effectively support clinicians workflows. The findings and simulation presented here reinforce the need for cross-cutting initiatives that target each level of interoperability to ensure that data are widely available to patients and physicians and that the data are of high quality. The Centers for Medicare & Medicaid Services’ Request for Information on the Health Technology Ecosystem, released in May 2025, indicate policymakers’ continued interest in revamping policy supporting interoperability, and the evolution and impact of those efforts deserve continued monitoring.^23^

Variation in ideal interoperability experiences by data type suggests that when addressing different levels of interoperability, technical solutions and policies also need to consider and address challenges specific to different data types. For example, we observed the lowest rate of ideal interoperability experiences for laboratory results, perhaps because laboratory standards are particularly complex, laboratory results involve multiple systems, and oversight over laboratory data is not centralized.^24,25,26^ In contrast, relatively high levels of ideal interoperability for immunizations may reflect the relative simplicity of immunization standards and the coordinating role of public health agencies and immunization information systems.

However, our results cannot differentiate between challenges related to data that are not standardized and therefore cannot be effectively used by receiving systems and systems that do not leverage standardized data to make it easy for physicians to use that information. There are limited policies incentivizing EHR usability for interoperable data, and the market to-date may have underinvested in this capability because the exchange of high-quality, standardized data was less common. It is possible that the increasing prominence of analytics and artificial intelligence may induce demand for higher-quality, standards-adherent data.^27^ Further work should monitor whether a reinforcing loop is emerging, wherein more widely available standardized data are driving demand for features that enhance usability, or whether a balancing loop leads to limited emphasis on data standardization and quality because EHRs lack features to leverage those standards and because investing in data quality results in externalities.

This collective action challenge indicates a need to consider additional effort to address data quality and the usability of exchanged data. The 21st Century Cures Act included several provisions designed to increase transparency into the performance of EHRs. Related technical tools, including a public tool to test adherence to standards, have also been developed,^28,29^ and there have been multiple recent efforts to push toward comprehensive and implementable metrics on data quality.^30,31^ These provisions might serve as a chassis to bring transparency to the extent of industry’s support for ideal interoperability experiences.

### Limitations

Our study has several limitations. First, it reflects the experience of an important group of clinicians—family physicians—but their perspectives may differ from those of other specialties, professions, and interested parties. Second, the survey instrument was not quantitatively validated against external criteria or constructs; however, our survey development process was grounded in in-depth interviews to ensure face and content validity. Third, results are subjective and reflect physician experience, which may evolve over time. Fourth, our results are cross-sectional and descriptive and not intended to indicate causation.

## Conclusions

In this cross-sectional study of family physicians, fewer than 15% of respondents reported ideal interoperability experiences—defined as often obtaining, finding, and reconciling external data within their EHRs—in 2024. Frequent poor experiences with interoperability among PCPs have implications for patient safety, quality, and costs. We did not find a consistent group that reported greater interoperability, and simulations examining the impact of policies and technical solutions demonstrate that actions need to target multiple levels of interoperability to make a demonstratable impact. Concerted efforts, aimed across the delivery system, should continue to improve the exchange of easily accessible and usable information for patients and clinicians.

## Notes

Supplement 1. eMethods. eTable 1. Respondent Characteristics eTable 2. Survey Response Related to Discrete Data Types eTable 3. Survey Response Related to Document Types eTable 4. Rates of Often or Sometimes Experiencing Ideal Interoperability for Multiple Data Types eTable 5. Multivariate Logistic Regression Predicting Ideal Interoperability for Discrete Data eTable 6. Multivariate Logistic Regression Predicting Ideal Interoperability for Documents

Supplement 2. Data Sharing Statement
