# Error in Figure 2

**DOI:** 10.1001/jamanetworkopen.2025.59209

**Published:** 2026-01-28

In the Original Investigation titled “EHR Interoperability Experiences Reported by Family Physicians,”^1^ published November 13, 2025, there was an error in Figure 2. The data for the Medications line and the Notes line were transposed for each variable. This article has been corrected.^1^
