# Supplementary material for: EHR Interoperability Experiences Reported by Family Physicians
Source: JAMA Netw Open. 2025 Nov 13;8(11):e2542460. doi: 10.1001/jamanetworkopen.2025.42460 (PMC13383672; doi:10.1001/jamanetworkopen.2025.42460)
Supplement: Supplement 1. — eMethods. eTable 1. Respondent Characteristics eTable 2. Survey Response Related to Discrete Data Types eTable 3. Survey Response Related to Document Types eTable 4. Rates of Often or Sometimes Experiencing Ideal Interoperability for Multiple Data Types eTable 5. Multivariate Logistic Regression Predicting Ideal Interoperability for Discrete Data eTable 6. Multivariate Logistic Regression Predicting Ideal Interoperability for Documents [file jamanetwopen-e2542460-s001.pdf]

Supplemental Online Content

Everson J, Adler-Milstein J, Phillips RL, Bazemore AW, Patel V. EHR interoperability experiences reported by family physicians. *JAMA Netw Open*. 2025;8(11):e2542460. doi:10.1001/jamanetworkopen.2025.42460

eMethods.

eTable 1. Respondent Characteristics

eTable 2. Survey Response Related to Discrete Data Types

eTable 3. Survey Response Related to Document Types

eTable 4. Rates of Often or Sometimes Experiencing Ideal Interoperability for Multiple Data Types

eTable 5. Multivariate Logistic Regression Predicting Ideal Interoperability for Discrete Data

eTable 6. Multivariate Logistic Regression Predicting Ideal Interoperability for Documents

This supplemental material has been provided by the authors to give readers additional information about their work.

© 2025 Everson J et al. *JAMA Network Open*.

eMethods

Technical Details on the Simulation of Potential Impact of Interventions to Improve Interoperability of Medication Information.

In the isolated improvement case, physicians that reported "sometimes," or "rarely," to each survey item about Medications were randomly assigned to instead have reported "Often" for that item. No other responses were altered for that individual. To achieve ideal interoperability, that individual would have needed to respond "often" to the questions not randomly reassigned. This procedure was repeated 1000 times and the median change in reported interoperability is reported on Table 3. In cases when more than one improvement was made, individual respondents were randomly assigned to the "often" category on both items. This reassignment was only applied to individuals initially reporting "sometimes" or "rarely" because respondents reporting "never" to the question about obtaining medications did not receive the other questions about medications and we sought to apply a consistent rule across the three items.

In the inter-related improvement case, we first calculated the probability that individuals who reported "Often" automatically obtaining information also reported "Often" easily finding information and that individuals that reported "Often" automatically obtaining information and Often easily finding information reported Often easily reconciling information. We retained these probabilities and then directly altered the percent of physicians that responded "Often" to each item and applied the observed transition probabilities to this increased baseline. For example, 67% of physicians that reported often automatically obtaining medications reported often easily finding those medications. Therefore, when we increased the percent of physicians that often automatically obtained medications from 32% as reported in the survey to 52%, the percent that would both often obtain medications and easily find them increases to 51.5*67=34.6%.

© 2025 Everson J et al. *JAMA Network Open*.

Appendix Table 1. Respondent Characteristics

	N = 8,122	%
**EHR System**		
eClinicalWorks	813	10
Allscripts	280	3.5
Cerner	552	6.8
Epic	3,428	42.2
Greenway	97	1.2
NextGen	328	4
Other	1,561	19.2
Practice Fusion	165	2.03
Unknown	130	1.6
athenaHealth	768	9.5
**Site Size**		
fa1-5 Providers	3,414	42
6-20 Providers	2,669	32.9
>20 Providers	2,039	25.1
**Main Site**		
Academic health center / faculty practice	612	7.5
Governmental	1,258	15.5
Hospital / Health System	2,840	35
Independently owned medical practice	2,336	28.8
Other	1,076	13.3
**Gender**		
Female	3,737	46.0
Male	4,330	53.3
Other/ prefer not to answer	55	0.7
**Age**		
Respondent Over 50	4,869	60.0
Respondent Under 50	3,253	40.1
**Location**		
Urban	6,884	15.2
Rural	1,238	84.8
**Percent of Panel Vulnerable Patients**		
0-10%	3,421	42.1
10-49%	2,914	35.9
>50%	1,787	22.0
**Value-Based Payment Participant**		
No	1,117	13.8
Yes	5,609	69.1
I don't know	1,396	17.2

© 2025 Everson J et al. *JAMA Network Open*.

Appendix Table 2. Survey Response Related to Discrete Data Types.

		Medications	Allergies	Problems	Immunizations (from all potential sources including state immunization registry)		Test results from commercial labs	Test results from outside hospitals or health systems
		Percent	Percent	Percent	Percent		Percent	Percent
**I automatically electronically obtain this information from outside organizations in my EHR**	Don't know	9	11	9	10	**I automatically electronically obtain this information from outside organizations in my EHR**	7	7
	Never	18	25	23	20		18	23
	Rarely	11	14	12	13		12	15
	Sometimes	30	26	32	29		27	36
	Often	32	23	23	28		33	19
	Not applicable						3	
**When I automatically electronically obtain this information from outside organizations, my EHR makes it easy to find**	Don't know	2	2	2	2	**When I automatically electronically obtain this information from outside organizations, my EHR makes it easy to find**	2	2
	Never	3	3	3	3		4	4
	Rarely	14	17	17	17		16	20
	Sometimes	44	43	48	41		44	52
	Often	38	35	30	37		33	22
**My EHR makes it easy to reconcile the differences between information from outside organizations and our internal data**	Don't know	4	5	4	5	**My EHR makes it easy to compare the results from outside organizations and our internal results**	4	3
	Never	9	7	8	7		21	16
	Rarely	19	18	18	18		23	26
	Sometimes	43	41	45	39		37	41
	Often	26	30	25	31		21	14
	Don't know					**When I automatically electronically obtain this information from outside organizations, it includes labs ordered by other physicians for my patients**	2	3
	Never						12	16
	Rarely						17	26
	Sometimes						45	41
	Often						24	15

Note: N=4,107 for all data types except test results from outside hospitals or health systems, N=4,006. For each data type, respondents skipped questions on whether information was easy to find if they reported that they did not know if they obtained that data in their EHR or never obtained it in their EHR. Percentages for the second and third question do not include these respondents in the denominator.

© 2025 Everson J et al. *JAMA Network Open*.

Appendix Table 3. Survey Response Related to Document Types.

		Independent Practice Notes	Health System Notes	Encounters	Independent Imaging Center Reports	Health system Imaging Reports
		Percent	Percent	Percent	Percent	Percent
**I automatically electronically obtain this document from outside organizations in my EHR**	Don't know	6	6	5	7	7
	Never	20	18	15	22	18
	Rarely	19	16	11	16	15
	Sometimes	37	41	36	29	36
	Often	19	19	32	26	24
**When I obtain this document from outside organizations (automatically or not), it is easy to find the document in my EHR**	Don't know	6	6	6	7	7
	Never	1	1	1	2	1
	Rarely	15	13	10	12	12
	Sometimes	49	51	48	43	47
	Often	29	28	35	36	34
**Within the document, it is easy to find the information that I'm looking**	Don't know	7	6	6	7	7
	Never	1	1	1	1	1
	Rarely	13	12	11	9	9
	Sometimes	51	52	50	41	45
	Often	29	28	32	42	38
N		4,015	4,022	4,022	3,991	4,015

Note: For each document type, respondents skipped questions on whether information was easy to find if they reported that they did not know if they obtained that document in their EHR or never obtained it in their EHR. Percentages for the second and third question do not include these respondents in the denominator.

© 2025 Everson J et al. *JAMA Network Open*.

Appendix Table 4. Rates of Often or Sometimes Experiencing Ideal Interoperability for Multiple Data Types.

	Often or sometimes automatically obtain in EHR	And often or sometimes easily find information	And often or sometimes easy to reconcile differences	
**Medications (Ref) (N=4,107)**	61.5	54.5	45.1	
**Allergies (N=4,107)**	49.7***	45.1***	40.4**	
**Problems (N=4,107)**	55.2***	49.3***	43.4***	
**Immunizations (from all potential sources including state immunization registry) (N=4,107)**	57.6***	51.3***	45	
	**Often or sometimes automatically obtain In EHR**	**And often or sometimes easy to find information**	**And often or sometimes obtain information ordered by other physicians for my patients**	**And often or sometimes easy to compare results from outside organizations**
**Test results from commercial labs (Ref) (N=4,107)**	60.1	51.4	42.2	31.9
**Test results from outside hospitals or health systems (N=4,006)**	54.6***	47.4***	44.9**	35.2***
	**Often or sometimes automatically obtain in EHR**	**And often or sometimes easy to find document in EHR**	**And often or sometimes easy to find information within the document**	
**Primary care notes / specialist consult reports from independent practices (Ref) (N=4,015)**	55.9	51.6	50.0	
**Health system primary care notes / specialist consult reports (N=4,022)**	60.1***	55.9***	54.3***	
**Encounters (N=4,022)**	67.9***	64.1***	61.4***	
**Independent imaging center reports (N=3,991)**	55.4	50.3	49.0	
**Health system imaging reports (N=4,015)**	60.4***	55.8***	54.6***	

Note: Each column indicates the percent of respondents that responded often to that question *and* prior questions about that data type. For each data and document type, respondents skipped questions on whether information was easy to find and use if they reported that they did not know if they obtained that information in their EHR or never obtained it in their EHR. Respondents indicating "Don't know" or "Never" were coded as not often automatically obtaining information in their EHR and retained in the denominator.
* $p<0.05$; ** $p<0.01$; *** $p<0.001$

© 2025 Everson J et al. *JAMA Network Open*.

Appendix Table 5. Multivariate Logistic Regression Predicting Ideal Interoperability for Discrete Data.

	Ideal Interoperability for Medications		Ideal Interoperability for Allergies		Ideal Interoperability for Problems		Ideal Interoperability for Test results from commercial labs		Ideal Interoperability for Test results from outside hospitals or health systems	
	Odds Ratio	95% Confidence Interval	Odds Ratio	95% Confidence Interval	Odds Ratio	95% Confidence Interval	Odds Ratio	95% Confidence Interval	Odds Ratio	95% Confidence Interval
**EHR System (Ref: athenaHealth)**										
Allscripts	0.23**	(0.09 - 0.59)	0.59	(0.27 - 1.30)	0.97	(0.49 - 1.93)	0.92	(0.44 - 1.95)	1.34	(0.66 - 2.74)
Cerner	0.72	(0.43 - 1.20)	0.93	(0.52 - 1.65)	0.70	(0.37 - 1.34)	0.71	(0.35 - 1.44)	0.43+	(0.18 - 1.03)
Epic	1.60**	(1.14 - 2.24)	3.15**	(2.15 - 4.62)	2.97**	(2.01 - 4.37)	1.46+	(0.95 - 2.26)	1.73*	(1.10 - 2.74)
Greenway	0.17+	(0.02 - 1.27)	-		0.34	(0.05 - 2.60)	1.32	(0.44 - 4.00)	1.17	(0.34 - 4.09)
NextGen	0.34**	(0.16 - 0.74)	0.33*	(0.13 - 0.86)	0.26*	(0.09 - 0.75)	1.14	(0.58 - 2.25)	1.45	(0.73 - 2.86)
Other	0.56**	(0.38 - 0.83)	0.74	(0.47 - 1.15)	0.69	(0.44 - 1.08)	0.85	(0.53 - 1.36)	0.85	(0.51 - 1.41)
Practice Fusion	0.47+	(0.21 - 1.05)	0.41	(0.14 - 1.20)	0.42	(0.14 - 1.22)	0.56	(0.21 - 1.52)	0.48	(0.14 - 1.64)
Unknown	0.67	(0.29 - 1.56)	1.50	(0.68 - 3.29)	1.15	(0.49 - 2.73)	1.16	(0.46 - 2.93)	1.55	(0.61 - 3.99)
eClinicalWorks	0.37**	(0.23 - 0.61)	0.60+	(0.36 - 1.02)	0.66	(0.40 - 1.12)	0.99	(0.59 - 1.64)	0.68	(0.38 - 1.24)
**Site Size (Ref: 1-5 Providers)**										
6-20 Providers	0.76*	(0.60 - 0.96)	0.78*	(0.62 - 0.98)	0.74*	(0.58 - 0.93)	0.73*	(0.55 - 0.98)	0.86	(0.64 - 1.16)
>20 Providers	0.79+	(0.61 - 1.02)	0.90	(0.70 - 1.16)	0.84	(0.65 - 1.09)	0.88	(0.64 - 1.21)	0.91	(0.66 - 1.27)
**Main Site (Ref: Hospital / Health System)**										
Academic health center / faculty practice	0.93	(0.64 - 1.35)	0.80	(0.55 - 1.15)	0.56**	(0.37 - 0.85)	0.78	(0.45 - 1.36)	0.70	(0.40 - 1.22)
Government Supported	0.83	(0.58 - 1.18)	0.95	(0.66 - 1.35)	1.01	(0.70 - 1.45)	1.04	(0.68 - 1.61)	0.96	(0.61 - 1.49)
Independently owned medical practice	1.15	(0.86 - 1.54)	1.16	(0.86 - 1.56)	1.14	(0.84 - 1.54)	1.56*	(1.10 - 2.20)	1.19	(0.83 - 1.71)
Other	0.84	(0.61 - 1.16)	0.96	(0.71 - 1.31)	0.95	(0.69 - 1.31)	1.27	(0.85 - 1.88)	1.28	(0.87 - 1.89)
**Gender (Ref: Female)**										
Male	0.85+	(0.70 - 1.03)	0.78*	(0.65 - 0.94)	0.80*	(0.66 - 0.97)	1.20	(0.95 - 1.53)	1.30*	(1.01 - 1.66)
Other/ prefer not to answer	-		-		-		0.62	(0.08 - 4.68)	0.77	(0.10 - 5.80)
Respondent Under 50 (Ref: 50+)	1.02	(0.84 - 1.23)	0.97	(0.80 - 1.17)	0.89	(0.73 - 1.09)	1.08	(0.85 - 1.38)	0.89	(0.69 - 1.14)
Rural (Ref: Urban)	0.63**	(0.46 - 0.86)	0.65**	(0.47 - 0.89)	0.53**	(0.37 - 0.74)	1.03	(0.73 - 1.45)	0.97	(0.68 - 1.39)

© 2025 Everson J et al. *JAMA Network Open*.

**Percent of Panel Vulnerable Patients (Ref: 0-10%)**										
10-49%	0.81+	(0.66 - 1.01)	0.84+	(0.68 - 1.03)	0.84	(0.67 - 1.04)	0.68**	(0.52 - 0.89)	0.84	(0.64 - 1.10)
>50%	0.96	(0.73 - 1.27)	0.81	(0.61 - 1.07)	0.96	(0.72 - 1.29)	0.92	(0.66 - 1.29)	0.91	(0.63 - 1.30)
**Value-Based Payment Participant (Ref: No)**										
I don't know	0.70+	(0.48 - 1.01)	0.88	(0.60 - 1.31)	1.01	(0.67 - 1.53)	0.62*	(0.39 - 0.98)	0.76	(0.46 - 1.24)
Yes	0.90	(0.67 - 1.23)	1.19	(0.85 - 1.66)	1.30	(0.91 - 1.86)	1.02	(0.71 - 1.45)	1.15	(0.77 - 1.72)
Constant	0.10**	(0.05 - 0.22)	0.06**	(0.03 - 0.12)	0.03**	(0.01 - 0.08)	0.08**	(0.03 - 0.20)	0.06**	(0.02 - 0.15)
Observations	4,081		4,044		4,069		4,107		4,006	

Note: Sample varies based on omission of variables that perfectly predicted the outcome.

95% CI in parentheses

© 2025 Everson J et al. *JAMA Network Open*.

Appendix Table 6. Multivariate Logistic Regression Predicting Ideal Interoperability for Documents.

	Ideal Interoperability for Independent Primary Care Notes / Specialist Consult Reports		Ideal Interoperability for Health System Primary Care Notes / Specialist Consult Reports		Ideal Interoperability for Encounters		Ideal Interoperability for Independent Imaging Center Reports		Ideal Interoperability for Health system Imaging Reports	
	Odds Ratio	95% Confidence Interval	Odds Ratio	95% Confidence Interval	Odds Ratio	95% Confidence Interval	Odds Ratio	95% Confidence Interval	Odds Ratio	95% Confidence Interval
**EHR System (Ref: athenaHealth)**										
Allscripts	0.72	(0.40 - 1.27)	1.03	(0.59 - 1.79)	0.99	(0.61 - 1.60)	0.43**	(0.26 - 0.72)	0.77	(0.48 - 1.22)
Cerner	0.56*	(0.32 - 0.99)	0.55*	(0.31 - 0.96)	0.73	(0.47 - 1.14)	0.30**	(0.18 - 0.51)	0.28**	(0.16 - 0.49)
Epic	0.79	(0.56 - 1.13)	1.01	(0.71 - 1.44)	1.29+	(0.95 - 1.74)	0.40**	(0.30 - 0.54)	0.58**	(0.43 - 0.78)
Greenway	1.20	(0.58 - 2.47)	1.06	(0.49 - 2.29)	0.73	(0.35 - 1.52)	0.44*	(0.21 - 0.91)	0.51+	(0.25 - 1.07)
NextGen	0.87	(0.51 - 1.48)	0.91	(0.53 - 1.56)	0.76	(0.47 - 1.24)	0.65+	(0.42 - 1.01)	0.71	(0.45 - 1.11)
Other	0.56**	(0.38 - 0.82)	0.69+	(0.46 - 1.02)	0.68*	(0.49 - 0.95)	0.40**	(0.29 - 0.55)	0.51**	(0.37 - 0.70)
Practice Fusion	0.61	(0.29 - 1.27)	0.75	(0.35 - 1.62)	0.63	(0.32 - 1.24)	0.41**	(0.22 - 0.75)	0.47*	(0.24 - 0.90)
Unknown	0.25+	(0.06 - 1.07)	0.29+	(0.07 - 1.24)	0.35+	(0.10 - 1.17)	0.06**	(0.01 - 0.42)	0.15*	(0.04 - 0.64)
eClinicalWorks	0.73	(0.48 - 1.10)	0.69+	(0.44 - 1.07)	0.74	(0.51 - 1.07)	0.53**	(0.38 - 0.75)	0.52**	(0.36 - 0.74)
**Site Size (Ref: 1-5 Providers)**										
6-20 Providers	0.88	(0.69 - 1.13)	0.83	(0.66 - 1.06)	0.92	(0.76 - 1.13)	0.82+	(0.66 - 1.03)	1.10	(0.88 - 1.36)
>20 Providers	0.76+	(0.57 - 1.01)	0.93	(0.71 - 1.21)	0.99	(0.80 - 1.24)	0.81+	(0.63 - 1.04)	1.09	(0.85 - 1.39)
**Main Site (Ref: Hospital / Health System)**										
Academic health center / faculty practice	0.67	(0.38 - 1.19)	0.56*	(0.34 - 0.92)	0.70*	(0.49 - 1.00)	0.90	(0.56 - 1.45)	0.91	(0.60 - 1.40)
Government Supported	1.48*	(1.02 - 2.14)	1.07	(0.75 - 1.51)	1.00	(0.75 - 1.33)	1.39+	(0.99 - 1.97)	1.30	(0.94 - 1.79)
Independently owned medical practice	1.71**	(1.26 - 2.33)	1.09	(0.81 - 1.47)	1.17	(0.91 - 1.50)	2.24**	(1.70 - 2.96)	1.96**	(1.50 - 2.57)
Other	1.55*	(1.09 - 2.20)	1.01	(0.72 - 1.41)	0.78+	(0.58 - 1.04)	1.64**	(1.19 - 2.27)	1.26	(0.92 - 1.74)
**Gender (Ref: Female)**										
Male	1.23+	(1.00 - 1.52)	1.08	(0.88 - 1.32)	0.97	(0.82 - 1.14)	0.91	(0.76 - 1.10)	1.08	(0.90 - 1.30)
Other/ prefer not to answer	0.98	(0.23 - 4.22)	0.70	(0.16 - 3.04)	0.40	(0.09 - 1.72)	0.65	(0.15 - 2.88)	0.31	(0.04 - 2.32)
Respondent Under 50 (Ref: 50+)	0.69**	(0.55 - 0.86)	0.80*	(0.65 - 0.99)	0.70**	(0.59 - 0.84)	0.89	(0.73 - 1.08)	0.75**	(0.62 - 0.91)
Rural (Ref: Urban)	1.02	(0.76 - 1.36)	0.92	(0.69 - 1.23)	0.99	(0.78 - 1.25)	0.74*	(0.56 - 0.98)	1.12	(0.87 - 1.43)

© 2025 Everson J et al. *JAMA Network Open*.

**Percent of Panel Vulnerable Patients (Ref: 0-10%)**										
10-49%	0.88	(0.70 - 1.11)	0.91	(0.73 - 1.14)	0.79*	(0.66 - 0.96)	0.79*	(0.64 - 0.98)	0.83+	(0.67 - 1.01)
>50%	0.66*	(0.48 - 0.91)	0.84	(0.62 - 1.13)	0.91	(0.72 - 1.16)	0.78+	(0.60 - 1.03)	0.93	(0.72 - 1.21)
**Value-Based Payment Participant (Ref: No)**										
I don't know	1.15	(0.76 - 1.73)	1.27	(0.84 - 1.93)	0.95	(0.67 - 1.35)	0.86	(0.60 - 1.23)	0.96	(0.67 - 1.39)
Yes	1.34+	(0.95 - 1.90)	1.50*	(1.05 - 2.15)	1.44*	(1.08 - 1.92)	1.19	(0.89 - 1.58)	1.42*	(1.05 - 1.92)
Constant	0.13**	(0.08 - 0.21)	0.13**	(0.08 - 0.22)	0.25**	(0.16 - 0.38)	0.33**	(0.21 - 0.51)	0.19**	(0.12 - 0.30)
Observations	4,015		4,022		4,022		3,991		4,015	

95% CI in parentheses
** p<0.01, * p<0.05, + p<0.1

© 2025 Everson J et al. *JAMA Network Open*.
