# Supplementary material for: EHR Interoperability Experiences Reported by Family Physicians
Source: JAMA Netw Open. 2025 Nov 13;8(11):e2542460. doi: 10.1001/jamanetworkopen.2025.42460 (PMC13383672; doi:10.1001/jamanetworkopen.2025.42460)
Supplement: Supplement 2. — Data Sharing Statement [file jamanetwopen-e2542460-s002.pdf]

Data Sharing Statement

Everson. EHR Interoperability Experiences Reported by Family Physicians. *JAMA Netw Open*. Published November 13, 2025. doi:10.1001/jamanetworkopen.2025.42460

Data
Data available: Yes
Data types: Deidentified participant data
How to access data: The data is available on request and completion of a collaboration agreement with the American Board of Family Medicine. More information is available at https://www.theabfm.org/research/external-collaborations/.
When available: With publication

Supporting Documents
Document types: None

Additional Information
Who can access the data: Data can be made available to researchers whose proposed use of the data has been approved.
Types of analyses: Data available for work focused on Family Medicine and healthcare quality.
Mechanisms of data availability: After approval of a proposal.
